# The effects of intravenous remifentanil on umbilical artery serum-derived exosomes in parturients undergoing epidural anesthesia: a randomized trail

**DOI:** 10.1186/s12884-023-05360-8

**Published:** 2023-01-14

**Authors:** Liangrong Wang, Juan Li, Xiaodan Yang, Yicheng Xiong, Zilu Wang, Li Li, Xinmiao Li, Hang Zhang, Yong Chen, Lina Lin, Xiangqing Xiong

**Affiliations:** 1 Department of Anesthesiology, the First Affiliated Hospital of Wenzhou Medical University, Shangcai Village, Nanbaixiang Town, Ouhai District, Wenzhou, Zhejiang Province 325000 China; 2 Women’s Hospital School Of Medicine Zhejiang University, Xueshi Road 1, Hangzhou, Zhejiang Province 310006, China; 3 Wenzhou Medical University, Chashan Higher Education Park, Wenzhou, Zhejiang Province 325035, China

**Keywords:** Repeated cesarean section, Remifentanil, Analgesia, Exosomes

## Abstract

**Background:**

Umbilical artery serum-derived exosomes (UEs) serve as messengers for maternal–fetal information exchange and cellular regulation. Intravenous remifentanil could be considered as an effective adjunct to epidural anesthesia in providing a favorable analgesia effect for cesarean section (C-section), but its effects on UEs are currently unknown.

**Methods:**

From 01/12/2021 to 30/06/2022, eligible parturients scheduled for repeated C-section at the First Affiliated Hospital of Wenzhou Medical University were randomized to receive either an intravenous bolus (0.15 μg/kg) followed by a continuous infusion (0.075 μg/kg/min) of remifentanil or normal saline throughout the procedure. The primary outcome was the number of UEs. Secondary outcomes included the size and protein amount of UEs, the vital signs, visceral pain score, sedation score, maternal satisfaction score, Apgar score, the incidence of neonatal asphyxia, umbilical arterial pH, and the presence of complications.

**Results:**

Nanoparticle tracking analysis indicated similar size of UEs between the two groups, but the number and protein amount of UEs were increased in the remifentanil group  compared to the control group (*P* < 0.05). In parturients receiving remifentanil, visceral pain scores were decreased, which was accompanied by the increased scores of maternal satisfaction with the anesthetic method (*P* < 0.05). Other maternal and neonatal outcomes were comparable between the two groups (*P* > 0.05).

**Conclusion:**

The intravenous administration of remifentanil increased the number of UEs in parturients undergoing repeated C-section under epidural anesthesia, with improved birth experience and minimal neonatal complications.

**Supplementary Information:**

The online version contains supplementary material available at 10.1186/s12884-023-05360-8.

## Background

China's "open childbirth policy" is anticipated to increase the number of women undergoing repeated cesarean section (C-section). C-section has typically been performed under epidural anesthesia due to its safety and controllability [1]. However, the preexisting abdominal adhesion and a scarred uterus considerably increase the painful response to visceral traction, diminish the efficacy of epidural anesthesia, and prolong the duration of the operation [2]. Moreover, visceral pain caused by incomplete anesthesia may further affect maternal childbirth experience [3]. As an ultrashort-acting μ receptor agonist that is rapidly metabolized without residual effect, remifentanil is widely recommended in anesthesia for obstetric surgery [4]. Continuous intravenous infusion of low-dose (0.05 μg/kg/min) remifentanil during epidural anesthesia has been shown to significantly improve the childbirth experience of parturients undergoing repeated C-cesarean without significant maternal or neonatal complications [1]. In the majority of studies, however, the safety of remifentanil in obstetric surgery has been questioned due to the increased incidence of hypoxemia associated with this agent [5]. To maximize the efficacy of epidural anesthesia while minimizing its adverse effects on maternal and neonatal outcomes, it is essential to investigate the rational use of remifentanil in repeat C-section.

The ability of extracellular vesicles (EVs) to transport specific components of proteins, lipids, RNA and DNA, as well as deliver various physiological information is well recognized [6]. Exosomes are endocytosis-originated, nanoscale (approximately 30–150 nm) EVs that are shed by most cell types and circulate in body fluids [7]. In a protective manner, maternal–fetal information sharing and cellular regulation occur intensively through the exchange of umbilical artery serum-derived exosomes (UEs), such as regulating normal pregnancy, maternal immunological function, and fetal and maternal vasculogenesis [8]. During C-section under epidural anesthesia, intravenous remifentanil provides a beneficial analgesic effect, but its effects on respiratory depression and hypoxia are also noticeable. Exosomes are sensitive to changes in cellular hypoxia, and hypoxia stimulates the production of exosomes in a variety of cells concurrently with the activation of the hypoxia inducible factor pathway [9]. However, the effects of intravenous remifentanil on UEs during repeated C-section are currently unknown.

Here, this prospective and randomized clinical trial was designed to investigate the effects of intravenous remifentanil in parturients undergoing repeated C-section under epidural anesthesia. The primary outcome was the number of UEs. Secondary outcomes included the size and protein amount of UEs, the vital signs, visceral pain score, sedation score, score of maternal satisfaction, Apgar score, the incidence of neonatal asphyxia, umbilical arterial pH, and the presence of complications.

## Methods

### Study setting

This prospective single-center and randomized clinical trial was approved by the ethical committee of The First Affiliated Hospital of Wenzhou Medical University on 21/09/2021 (Chairperson Pro. Jinglin Xia, NO.KY2021-119) and registered at chictr.org.cn (ChiCTR2100053635) on 26/11/2021. The study was conducted in the First Affiliated Hospital of Wenzhou Medical University between 01/12/2021 and 30/06/2022 according to the criteria of Declaration of Helsinki, and informed consent was obtained from each participant.

### Patient enrollment

Parturients with singleton, full-term pregnancies, ASA physical status I or II, age 20–40 years, height 150–170 cm, weight 50–80 kg, and body mass index (BMI) less than 35 kg/m^2^ were recruited. Exclusion criteria included the presence of hepatic and renal dysfunction, psychiatric disorders, contraindications for epidural anesthesia, epidural anesthesia failure, the requirement of general anesthesia, prolonged surgical duration (> 1.5 h), and long-term use of analgesics, sedatives, or antidepressants. Those who experienced perioperative hemorrhage, which was defined as a blood loss larger than 500 mL, were also removed.

### Randomization and group allocation

Using a simple randomization procedure (1:1 ratio, www.randomization.com), forty-four parturients were enrolled and randomly assigned into either the remifentanil group (Group R) or the control group (Group E). The allocations were sealed in opaque envelopes by the investigator LL and only opened prior to epidural anesthesia by the same attending anesthesiologist (LW) who was not involved with data collection. Epidural anesthesia was planned for the C-section in both two groups. In Group R, an intravenous bolus of 0.15 μg/kg remifentanil was administered for 10 s at skin incision, followed by a continuous infusion of remifentanil at a rate of 0.075 μg/kg/min throughout the procedure, whereas in Group E, the equal volume of normal saline was administered instead. Data collection was performed by JL, XY, ZW, XL, and HZ who were unaware of group allocation.

### Anesthetic procedure

No premedication was administered, and parturients were required to abstain from food and liquid intake for at least 6 h and 2 h before surgery, respectively. Intravenous access was established and nasal oxygenation at a rate of 3 L/min was administered upon entering the operating room. As a preload prior to anesthesia, 10 mL/kg of Ringer's lactate was administered. The parturient was placed in a left lateral position, and an 18-gauge cannula needle was used to puncture the epidural space, followed by the insertion of an epidural catheter. After administering 3 mL of 2% lidocaine as a test dose via the epidural catheter, an initial bolus of 6 mL 0.5% ropivacaine was delivered, followed by a supplemental dose of 6–9 mL of the same local anesthetic solution to ensure the upper sensory block level of T6. The local anesthetic solution could be administered as needed, up to a safe maximum dose of 200 mg. If the sensory block level failed to reach T6 at 30 min after epidural administration, intravenous analgesics as rescue agents or general anesthesia would be considered and the parturient would be excluded from the study. The patient-controlled epidural analgesia package containing 150 mg ropivacaine and 3 mg morphine in 100 mL normal saline, with a bolus of 2 mL, a background flow of 2 mL/h, and a lockout interval of 15 min, was initiated for postoperative analgesia before transfer to the post-anesthesia care unit (PACU).

### Vital signs recording

Mean arterial blood pressure (MAP), heart rate (HR), respiration rate (RR), and oxygen saturation (SpO_2_) were monitored before anesthesia (T0), at the skin incision (T1), peritoneum incision (T2), neonatal delivery (T3), placental delivery (T4), uterus closure (T5), and abdominal cavity closure (T6).

### Visceral pain score

Visceral pain was defined as pain associated with uterine exteriorization and peritoneal traction. The intensity of pain was measured at time points T1-6 using a standard visual analogue scale (VAS) ranging from 0 to 10.

### Evaluation of sedation

The sedation level of the parturient was determined using the Ramsay sedation score system, where level 1 indicates anxiety or irritability; level 2 indicates cooperation, quietness, and well orientation; level 3 indicates drowsiness but responsiveness to instructions; level 4 indicates a rapid response to tapping the brow or strong sound stimulation; level 5 indicates delayed response to tapping the brow or strong sound stimulation, and level 6 indicates no response to tapping the brow or strong sound stimulation.

### Maternal satisfaction with the anesthetic method

At 24 h postoperatively, maternal satisfaction with the anesthetic method was evaluated using a five-point scale (1 = completely dissatisfied, 2 = dissatisfied, 3 = neutral or undecided, 4 = satisfied, and5 = completely satisfied).

### Evaluation of Apgar scores and neonatal asphyxia

The neonatal Apgar scores were recorded at 1 min, 5 min, and 10 min after birth, and the occurrence of neonatal asphyxia was documented. Neonatal asphyxia was diagnosed using the following clinical criteria: neurological abnormalities or neonatal resuscitation required at birth, and/or an Apgar score < 7 at 5 min.

### Umbilical arterial pH and the isolation of exosomes

6 mL of blood samples were obtained from the umbilical artery at 5 min after delivery. One portion of each sample was analyzed for pH. UEs were isolated from another portion of umbilical cord sample using ExoQuick exosome precipitation solution (System Biosciences, Palo Alto, CA, USA), a commercially available kit that gently precipitates exosomes ranging in size from 30 and 200 nm [10]. Briefly, umbilical cord serum samples were centrifuged at 3000 g for 15 min, followed by the addition of 250 mL of ExoQuick exosome precipitation solution to 1 mL of the serum supernatants. The mixture was then refrigerated for 30 min and centrifuged at 1500 g for another 30 min. The residual solution was obtained and centrifuged at 1500 g for 5 min to remove the supernatant. The exosome pellet was resuspended in 500 mL of phosphate-buffered saline (PBS) and stored at -80 °C.

### Characterization of exosomes using nanoparticle tracking analysis (NTA)

NTA measurements were performed in a flow model using a NanoSight NS300 instrument (Malvern Panalytical, Malvern, United Kingdom) equipped with a 488 nm laser and sCMOS camera module (Malvern Panalytical, Malvern, United Kingdom). Each sample was subjected to NTA at least three times to calculate the mean values. To ensure the accuracy of results, all culture medium samples were identically diluted.

### Exosome validation by transmission electron microscopy (TEM)

The morphology of the exosome was identified using TEM. Briefly, a suspension of freshly isolated exosomes was dropped onto a formvar carbon-coated copper electron microscopy grid (Plano, Wetzlar Germany). The grid was then negatively stained for 1 min with 2% uranyl acetate solution, washed with PBS, and dried at room temperature. Finally, images were acquired with an EM 900 transmission electron microscope (Zeiss, Germany) at a voltage of 80-90kv.

### Exosomal biomarkers measurement

The total protein content of exosomes was extracted using a homemade cell lysis buffer, followed by protein precipitation using a 5 × protein loading buffer (ABM, Vancouver, Canada). After 5 min of heating in a water bath at 100 °C, the mixture was transferred to a polyvinylidene difluoride membrane (Millipore, Burlington, MA, USA). The PVDF membrane was blocked by incubating it for 1 h at room temperature in milk Tris-buffered saline with Tween20 solution. After that, the membrane was incubated for 15 h with primary antibodies against rabbit anti-mouse CD63 (1:1000; Abcam, USA) and tumor susceptibility gene 101 (TSG101; 1:1000; Abcam, USA). The membranes were then incubated for 1 h at room temperature with HRP-conjugated secondary antibodies (1:1500; Abcam, USA). Finally, the expression of proteins was determined using enhanced chemiluminescence reagents.

### Measurement of other outcomes

The adverse events that occurred during surgery and PACU stay were documented. Remifentanil infusion was stopped if the parturient developed respiratory depression, defined as SpO_2_ < 90% on room air or RR < 8 times/min, the patient was awakened, and ventilation was manually assisted. In addition, hypotension, bradycardia, shivering, cough, nausea, and vomiting were also recorded. (1) Hypotension was defined as a systolic blood pressure < 90 mmHg or a decrease in blood pressure of at least 30% when compared to baseline value, then fluid infusion was accelerated and/or a 0.25 mg bolus of phenylephrine was administered if necessary. (2) If the parturient developed bradycardia, which was defined as a heart rate of less than 60 beats per minute or a drop of more than 30% from baseline, 0.5 mg atropine was then intravenously injected. (3) A four-point scale was used to assess postoperative nausea and vomiting (PONV) (1 = no nausea, 2 = mild nausea, 3 = severe nausea, and 4 = vomiting) [11]. (4) Shivering was also assessed (0 for no shivering, 1 for mild fasciculations of the face or neck, 2 for moderate, visible tremor in more than one muscle group, and 3 for severe, gross muscular activity involving the entire body) [11]. (4) Major side effects of the remifentanil were also documented, including lightheadedness, dyspnea, blurred vision, chest pain, and muscle stiffness and tightness.

### Statistical analysis

The sample size was calculated using the OpenEpi software version 2.3.1 with the number of UEs as the primary outcome. In our preliminary study of 10 cases (n = 5 in each group), the mean number of UEs was 62 × 10^8^ in parturients receiving epidural anesthesia alone, and 99 × 10^8^ in parturients receiving additional remifentanil. We accepted a 5% significance level (two-tailed), an 80% power value, and a 10% dropout rate, resulting in a sample size of n = 22 for each group.

Statistical analysis was performed with SPSS 26.0 software (SPSS Inc, Chicago, IL, USA). The Shapiro–Wilk test was used to examine the normality of the data. Continuous and normally distributed data were reported as mean ± standard deviation (SD), and an independent t-test was performed to compare data between the two groups. Repeated measures analysis of variance was used to compare repeated measured data between different time points within each group, non-normally distributed data were log-normally transformed if necessary before adopting the above statistical methods. Data with non-normal distribution were expressed as median (interquartile range, IQR) and analyzed using the Mann–Whitney U test. Moreover, categorical data were expressed as numbers (%), and Fisher’s exact test was employed to compare these data between the two groups. Statistical significance was defined as *P* value less than 0.05.

## Results

### The demographic characteristics of the participants

**Fig. 1:**
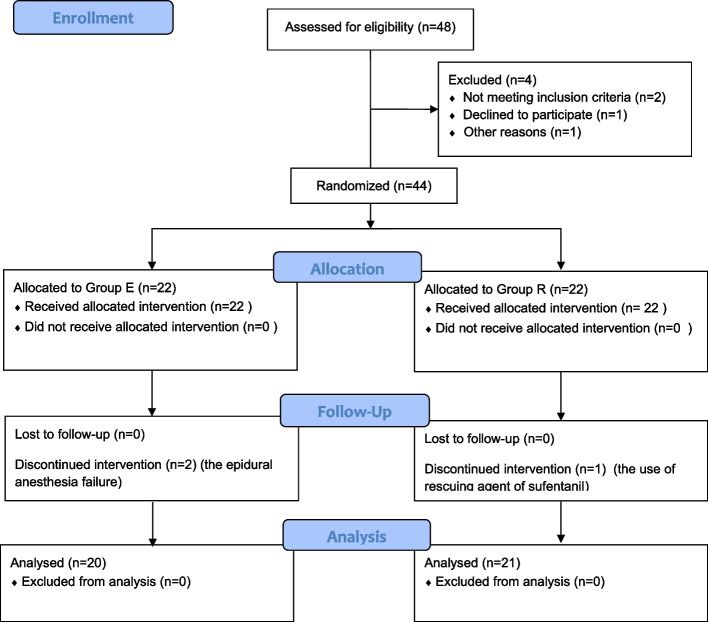
CONSORT flow chart diagram of the study. Image shows the CONSORT flowchart diagram of the study

As shown in Fig. 1, a total of 44 parturients were enrolled in the study, and 41 of them, including 20 cases in Group E and 21 cases in Group R, were included in the final analysis. Two cases were excluded due to the epidural anesthesia failure, and another case was excluded due to the use of sufentanil as a rescue agent during the surgery.

The demographic characterizations of the parturients in the two groups were comparable in terms of age, ASA physical status, weight, height, pregnant week, smoking history, alcohol drinking history, gestational weight gain, hypertensive disorders of pregnancy, gestational diabetes, surgery duration, the upper level of sensory block, the volume of anesthetic solution, the dose of oxytocin and the total volume of blood loss (*P* > 0.05, Table 1).

**Table 1:** The demographic characterizations of the parturients

Variables	Group E (*n* = 20)	Group R (*n* = 21)	*P* value
Age (year)	32.1 ± 3.8	31.8 ± 5.0	0.838 ^a^
ASA physical status (n, I/II)	10/10	13/8	0.758 ^b^
Weight (kg)	71.8 ± 7.1	70.1 ± 9.3	0.525 ^a^
Height (cm)	158.3 ± 3.7	159.2 ± 4.1	0.466 ^a^
BMI (kg/m^2^)	29.0 ± 2.6	27.6 ± 3.3	0.142 ^a^
Pregnant week (weeks)	38.8 ± 1.2	38.8 ± 1.1	0.899 ^a^
Smoking habit (n (%))	0 (0%)	0 (0%)	NA ^b^
Alcohol drinking habit (n (%))	0 (0%)	0 (0%)	NA ^b^
Gestational weight gain (kg)	13.4 ± 3.3	14.6 ± 3.8	0.275 ^a^
Hypertensive disorders of pregnancy (n (%))	2 (10%)	2 (9.5%)	0.678 ^b^
Gestational diabetes (n (%))	3 (15%)	4 (19%)	0.529 ^b^
Surgery duration (min)	51.6 ± 8.3	54.5 ± 9.0	0.287 ^a^
Upper sensory block level (T)	5 [4, 6]	5 [4, 6]	0.846 ^c^
Volume of anesthetic solution (mL)	14.1 ± 1.2	14.3 ± 1.3	0.545 ^a^
Total oxytocin dose (U)	20.5 ± 6.0	20.0 ± 5.5	0.783 ^a^
Volume of blood loss (mL)	300.0 ± 56.2	307.1 ± 57.6	0.690 ^a^

Data are expressed as mean ± standard deviation or median [interquartile range] or number (%)

*ASA* American Society of Anesthesiologists, *BMI* body mass index

^a^ Independent t-test; ^b^ Fisher’s exact test; ^c^ Mann–Whitney U test

### Alterations of UEs

**Fig. 2:**
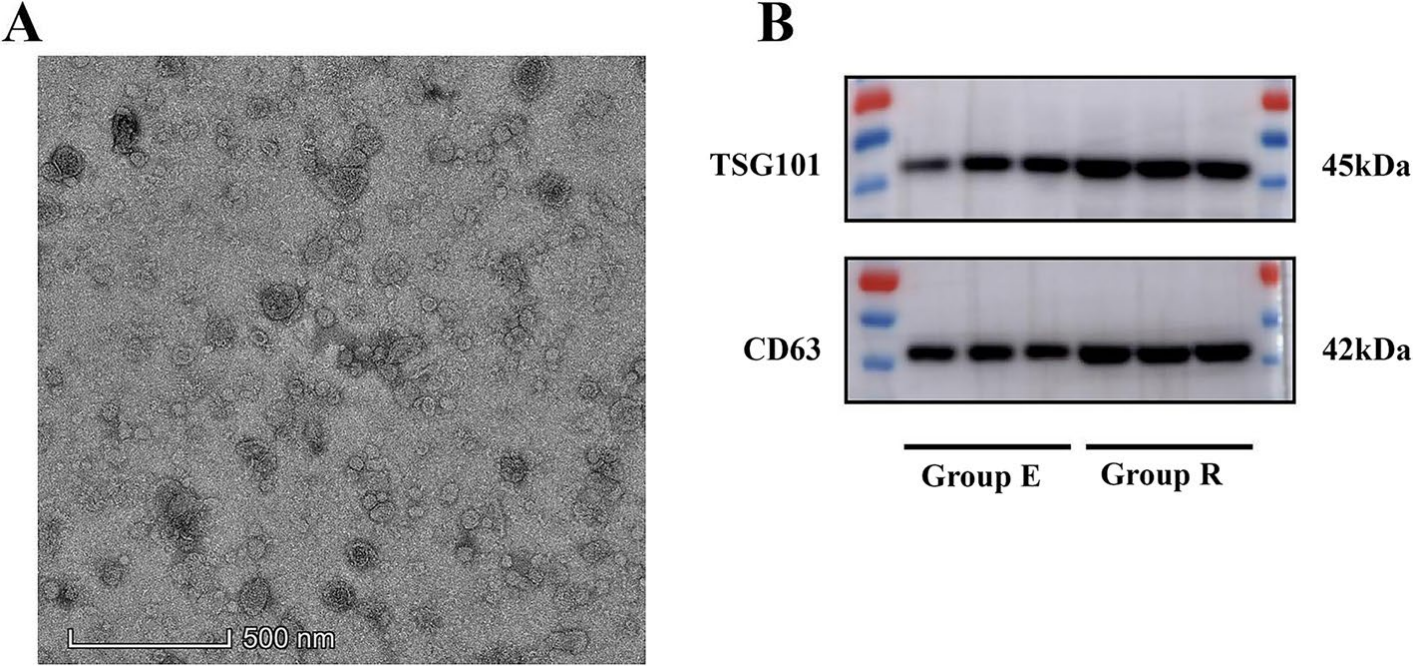
Characterisations of UEs. **A** UEs were analyzed by the transmission electron microscopy. **B** Expression of exosomal biomarkers CD63 and TSG101 were detected by western blotting. UEs, umbilical cord serum-derived exosomes; NTA, nanoparticle tracking analysis; TSG101, tumor susceptibility gene 101

The morphology and phenotype of exosomes were identified using TEM and NTA. It was noted that the particles displayed typical lipid bilayer membrane-encapsulated nanoparticles (Fig. 2A). The NTA assay revealed, as shown in Table 2 and Fig. 2B, that the diameters of the particles isolated from the umbilical cord serum were 80–130 nm, a typical size of exosomes. As shown in Table 2, the size of UEs did not differed significantly between the two groups (*P* > 0.05), the number and the total protein amount of UEs were increased in Group R compared to Group E (*P* < 0.05), but there was no significant difference in protein per particle between the two groups (*P* > 0.05), indicating that the increased number of UEs, but not the upregulated protein in each particle, contributed to the increased protein amount of UEs. Moreover, exosome biomarker proteins including CD63 and TSG101 were expressed in both groups (Fig. 2B).

**Table 2:** Characteristics of UEs by NTA

Variables	Group E (*n* = 20)	Group R (*n* = 21)	*P* value
Diameter (nm)	91.45 ± 17.14	93.30 ± 16.71	0.728 ^a^
Number of UEs (× 10^8^)	68.15 ± 32.19	97.44 ± 43.92	0.020 ^a^
Protein amount (μg)	147.38 ± 59.81	227.55 ± 112.97	0.008 ^a^
protein/particle (× 10^–9^)	9.9 [5.4, 14.5]	9.7 [5.4, 13.5]	0.855 ^b^

Data expressed as mean ± standard deviation or median [interquartile range]

*UEs* umbilical cord serum-derived exosomes, *NTA* nanoparticle tracking analysis

^a^ Independent t-test; ^b^ Mann–Whitney U test

### The vital signs of the parturients

**Fig. 3:**
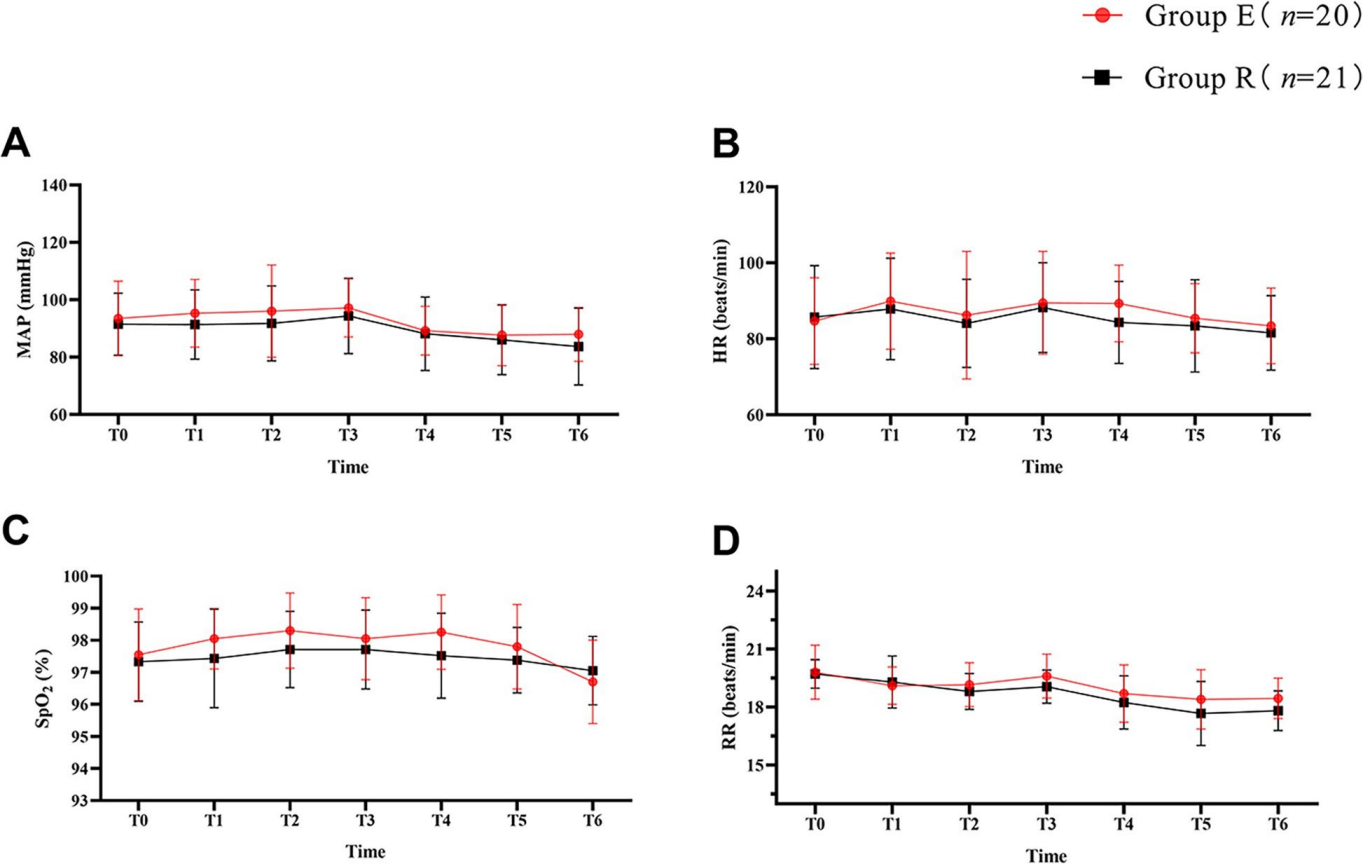
The vital signs of the parturients. T0 = immediately before anesthesia; T1 = skin incision; T2 = peritoneum incision; T3 = neonatal delivery; T4 = placental delivery; T5 = suture of the uterus; T6 = closure of the abdominal cavity; MAP, mean arterial blood pressure; HR, heart rate; SpO_2_, oxygen saturation; RR, respiration rate

As demonstrated in Fig. 3, no significant differences in blood pressure, heart rate, respiratory rate and oxygen saturation between the two groups were found (all *P* > 0.05).

### Visceral pain scores of the parturients

The parturients in Group R had significantly lower VAS scores associated with visceral traction at T2-6 than those in Group E (*P* < 0.05, Table 3).

**Table 3:** VAS scores of the parturients (log)

Group	T1	T2	T3	T4	T5	T6
Group E (*n* = 20)	0.14 ± 0.03	0.52 ± 0.14	0.66 ± 0.06	0.63 ± 0.11	0.51 ± 0.12	0.43 ± 0.12
Group R (*n* = 21)	0.14 ± 0.04	0.39 ± 0.09	0.48 ± 0.12	0.45 ± 0.12	0.37 ± 0.11	0.30 ± 0.18
*P* value	0.940 ^a^	0.001 ^a^	< 0.001 ^a^	< 0.001 ^a^	< 0.001 ^a^	0.006 ^a^

Data are expressed as mean ± standard deviation

*VAS* visual analogue scale, *T1* skin incision, *T2* peritoneum incision, *T3* neonatal delivery, *T4* placental delivery, *T5* suture of the uterus, *T6* closure of the abdominal cavity

^a^repeated measures ANOVA

### Ramsay scores of the parturients

Compared to group E, the Ramsay scores of the parturients were significantly increased at T3-T6 in group R (*P* < 0.05, Table 4).

**Table 4:** Ramsay Scores of the parturients (log)

Group	T1	T2	T3	T4	T5	T6
Group E (*n* = 20)	0.45 ± 0.12	0.42 ± 0.07	0.47 ± 0.05	0.45 ± 0.06	0.49 ± 0.07	0.49 ± 0.05
Group R (*n* = 21)	0.46 ± 0.06	0.44 ± 0.07	0.52 ± 0.05	0.52 ± 0.06	0.55 ± 0.05	0.55 ± 0.06
*P* value	0.087 ^a^	0.298 ^a^	0.003^a^	0.001 ^a^	0.003 ^a^	0.001 ^a^

Data are expressed as mean ± standard deviation

*T1* skin incision, *T2* peritoneum incision, *T3* neonatal delivery, *T4* placental delivery, *T5* suture of the uterus, *T6* closure of the abdominal cavity

^a^repeated measures ANOVA

### The parturients’ satisfaction and the incidence of adverse outcomes

The parturients in Group R had significantly greater satisfaction scores with anesthetic technique than Group E (*P* < 0.05), and both the incidence and severity of shivering and PONV during surgery and PACU stay were comparable between the two groups (*P* > 0.05). Similarly, the incidence of other adverse outcomes, which included hypotension, bradycardia, respiratory depression and PONV, did not show significant differences between the two groups during surgery and PACU stay (*P* > 0.05, Table 5).

**Table 5:** The parturients’ satisfaction and clinical outcomes

Variables	Group E (*n* = 20)	Group R (*n* = 21)	*P* value
Satisfaction score	4 [3, 4]	4 [4, 5]	0.015 ^a^
Hypotension (n (%))	3 (15%)	5 (23.8%)	0.697 ^b^
Bradycardia (n (%))	0 (0%)	0 (0%)	NA ^b^
PONV (n (%))	5 (25%)	4 (19%)	0.719 ^b^
PONV score	1 [1, 1.75]	1 [1, 1]	0.678 ^a^
Shivering (n (%))	4 (20%)	5 (28.6%)	> 0.999 ^b^
Shivering score	0 [0, 0]	0 [0, 0.5]	0.692 ^a^
Agent-related side effects (n (%))	0 (0%)	0 (0%)	NA ^b^

Data are expressed as median [interquartile range] or number (%)

*PONV* postoperative nausea and vomiting

^a^Mann-Whitney U test; ^b^Fisher’s exact test

### Neonatal outcomes

As shown in Table 6, no significant differences were found between the two groups in terms of the overall incidence of neonatal asphyxia, the number of the newborns who required neonatal resuscitation at birth, Apgar scores at 1,5 and 10 min after birth, pH values, and lactic acid levels in umbilical artery (*P* > 0.05).

**Table 6:** Neonatal outcomes

Variables	Group E (*n* = 20)	Group R (*n* = 21)	*P* value
Neonatal asphyxia (n (%))	0 (0%)	0 (0%)	NA ^a^
Apgar score at 1 min	9 [9]	9 [9, 10]	0.783 ^b^
Apgar score at 5 min	9 [9, 9.5]	9.5 [9, 10]	0.651 ^b^
Apgar score at 10 min	10 [10]	10 [10]	0.306 ^b^
PH value of umbilical artery	7.35 ± 0.03	7.33 ± 0.05	0.128 ^c^
Lactic acid levels in umbilical artery (mmol/L)	1.43 ± 0.30	1.63 ± 0.41	0.087 ^c^

Data are expressed as mean ± standard deviation or median [interquartile range]

^a^Fisher’s exact test; ^b^ Mann–Whitney U test; ^c^ Independent t-test

## Discussion

The primary finding of our present study was that the intravenous administration of remifentanil increased the number of UEs in parturients undergoing repeated C-section under epidural anesthesia. As expected, intravenous remifentanil might be considered as an effective adjunct to epidural ropivacaine for the relief of visceral pain and improvement of childbirth experience in parturients, with no significant adverse effects in neonates.

Visceral pain is described as a dull, aching, ill-defined, and unpleasant feeling that is poorly localized and appears to come from deep within the body, and is frequently accompanied by malaise and strong autonomic reflexes [12]. The incidence of visceral pain ranges from 10 to 50% in parturients undergoing epidural anesthesia during peritoneal traction or uterine rotation, which is more common in parturient undergoing repeated C-section [13]. To provide optimal anesthesia care for this population of parturients, the supplemental intravenous agents with minimal effects on neonate and parturient should be considered. Opioids are well-known to depress C fiber-mediated sympathetic reflexes and share synergistic interaction with general anesthetic agents, making them promising therapeutic strategies for the attenuation of visceral pain [14].

Remifentanil, a commonly used analgesic in obstetric anesthesia, could provide effective analgesia during monitored anesthesia care in a general patient population with minimal effects on respiration and hemodynamics at dose of 0.1 μg/kg/min [15]. And similar outcomes were observed in parturients who were continuously administered 0.05 μg/kg/min remifentanil [1]. For repeated C-section parturients who require intensive analgesia during surgical procedures, the dosage and method of remifentanil infusion are of the utmost importance. However, remifentanil is thought to rapidly and extensively cross the placenta, and its concentration in fetal blood is theoretically close to the maternal level [4, 16]; thus, the increased dose may result in varied neonatal outcomes, with respiratory depression being the most prevalent adverse effect during C-section. Our findings demonstrated that an intravenous bolus of 0.15 μg /kg followed by a continuous infusion of 0.075 μg/kg/min remifentanil was associated with minimal neonatal outcomes. Nonetheless, an intravenous bolus of 0.5 μg/kg followed by a continuous infusion of 0.2 μg/kg/min remifentanil was reported to cause partial newborn depression that required brief manual ventilation during general anesthesia with propofol for planned C-section [17]. Moreover, a bolus administration of 1 μg/kg remifentanil before general anesthesia induction would cause transient but significantly increased risk of neonatal respiratory depression during the first minute after caesarean delivery [18]. Importantly, though no significant differences were found between the two groups in regard to neonatal outcomes based on the sample size calculated with the number of UEs as the primary outcome, lactic acid levels in Group R tended to be higher than those in Group E (p = 0.087), which might achieve statistical significance with a larger sample size, therefore the effects of prolonged intravenous remifentanil administration on neonatal outcomes should be carefully considered.

Due to its rapid metabolism, redistribution, or both, the neonatal concentration may not reach a sufficient level to induce fatal neonatal respiratory depression [19]. Pretreatment with remifentanil can reduce the effects of oxidative stress and protect cells from hypoxia-induced senescence and necrosis, according to basic experiments [9, 20]. As messengers between mother and fetus, UEs not only promote cell proliferation and migration, but also serve as biomarkers of fetal health and nutritional status [8]. Since exosomes can function as messengers between parent and recipient cells, they may be involved in cell-to-cell and organ-to-organ communication in metabolic diseases [21], and obesity would undoubtedly affect the content of exosomes [22]. However, the commonly concomitant metabolic statuses and diseases associated with obesity were comparable between our two groups, the alteration in the number of UEs was therefore primarily attributed to remifentanil administration. In support of this, a recent study showed that morphine exposure led to a significant increase in astrocyte-derived EVs release without affecting the size distribution of these EVs [23]. The in utero and postnatal oxycodone administration increases the size of brain-derived EVs in rats, but has no significant effect on their number [24]. Our study showed that an intravenous bolus of 0.15 μg/kg followed by a continuous infusion of 0.075 μg/kg/min remifentanil during C-section increased the number and protein amount of UEs without affecting their size distribution. Given the oxygen sensitivity of exosomes, the increase in the number and protein content of UEs observed in this study may be an adaptive response to the imperceptible induction in the fetal oxygen saturation caused by remifentanil.

Moreover, the Ramsay scores of the parturients who received remifentanil were increased significantly, and no subject developed respiratory depression (RR < 8 times/ min) without impairments in oxygen saturation and other vital signs. Continuous intravenous administration of remifentanil had little effect on uterine contraction, given that the dose of oxytocin administration and the total volume of blood loss were comparable between two groups. And, consistent with previous studies, no significant adverse effects on neonates were reported [1, 25]. These findings suggested that this intravenous remifentanil administration protocol would be safe and effective as an adjunct to epidural anesthesia.

Up to 85% of parturient undergoing C-section may experience shivering after spinal anesthesia due to an impairment of thermoregulation [26]. It has been demonstrated that shivering is more common after administration of short-acting opioids, and the dose of remifentanil is positively correlates with the incidence of shivering [27]. Our present study revealed that remifentanil did not significantly affect the incidence and severity of shivering in parturients during surgery and PACU stay. This discrepancy may be explained by the fact that the total dose of remifentanil was relatively low, and the results might vary over a longer period of time [28]. This study did not report any other adverse effects associated with remifentanil administration, of which dizziness, blurred vision, chest pain, and muscle stiffness and tightness are the most common.

Several limitations should be addressed. First, the maximum safe dose of intravenous remifentanil for C-section under epidural anesthesia is undetermined, and we should be aware that adverse outcomes are significant with bolus dose larger than 40 ug or the concomitant use of long-acting opioids [29, 30]. Second, the parturients enrolled in our study were overweighted, even though there was no significant difference in the pharmacokinetics of remifentanil between obese and lean subjects [31], the results might be more convincing if the dose of remifentanil was calculated by the ideal rather than the actual body weight of the participants. Third, intravenous administration of remifentanil resulted in partial changes in the UEs phenotype in parturients undergoing C-section in this study, the changes of UEs induced by higher doses of remifentanil warrant further study. Finally, the long-term maternal and neonatal effects of adding intravenous remifentanil to epidural anesthesia need to be clarified.

## Conclusion

An intravenous bolus of 0.15 μg/kg followed by a continuous infusion of 0.075 μg/kg/min remifentanil increased the number of UEs in parturients undergoing repeated C-section under epidural anesthesia, effectively reduced visceral pain and improved birth experience of the parturients, without causing significant neonatal complications.

## Supplementary Information

**Additional file 1.**

**Additional file 2.**

## Data Availability

The datasets generated and analyzed during the current study are available from the corresponding author, on reasonable request.

## Abbreviations

C-section: Cesarean section
Evs: Extracellular vesicles
UEs: Umbilical artery serum-derived exosomes
BMI: Body mass index
PACU: Post-anesthesia care unit
MAP: Mean arterial blood pressure
HR: Heart rate
RR: Respiration rate
SpO_2_: Oxygen saturation
VAS: Visual analogue scale
PBS: Phosphate-buffered saline
NTA: Nanoparticle tracking analysis
TEM: Transmission electron microscopy
PONV: Postoperative nausea and vomiting
SD: Standard deviation
IQR: Interquartile range
