# Correction: The effects of intravenous remifentanil on umbilical artery serum-derived exosomes in parturients undergoing epidural anesthesia: a randomized trial

**DOI:** 10.1186/s12884-023-05401-2

**Published:** 2023-02-01

**Authors:** Liangrong Wang, Juan Li, Xiaodan Yang, Yicheng Xiong, Zilu Wang, Li Li, Xinmiao Li, Hang Zhang, Yong Chen, Lina Lin, Xiangqing Xiong

**Affiliations:** 1 Department of Anesthesiology, the First Affiliated Hospital of Wenzhou Medical University, Shangcai Village, Nanbaixiang Town, Ouhai District, Wenzhou, 325000 Zhejiang Province China; 2 Women’s Hospital School of Medicine Zhejiang University, Xueshi Road 1, Hangzhou, 310006 Zhejiang Province China; 3 Wenzhou Medical University, Chashan Higher Education Park, Wenzhou, 325035 Zhejiang Province China

**Correction: BMC Pregnancy Childbirth 23, 29 (2023)**

**https://doi.org/10.1186/s12884-023-05360-8**

Following publication of the original article [1], the author reported that in the article the title "trial" was incorrectly written as "trail". In addition, Dr. Xiangqing Xiong's affiliation is only “the First Affiliated Hospital of Wenzhou Medical University” and is presented correctly in this correction article.

The original article [1] has been corrected.
