# Supplementary figures and images for: The effects of intravenous remifentanil on umbilical artery serum-derived exosomes in parturients undergoing epidural anesthesia: a randomized trail
Source: BMC Pregnancy Childbirth. 2023 Jan 14;23:29. doi: 10.1186/s12884-023-05360-8 (PMC9840320; doi:10.1186/s12884-023-05360-8)

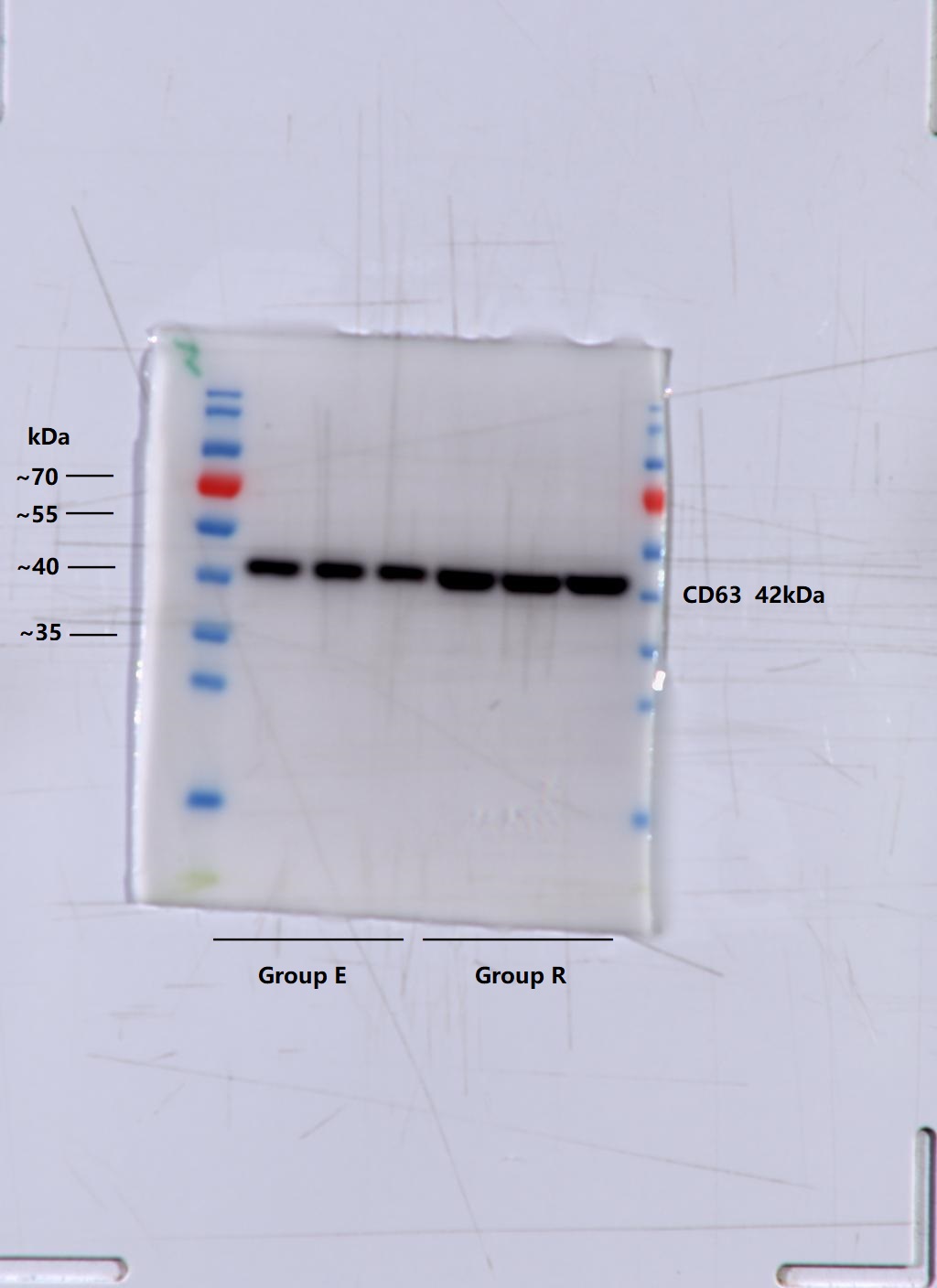

Supplement: Supplementary file 1 — Additional file 1. [file 12884_2023_5360_MOESM1_ESM.jpg]

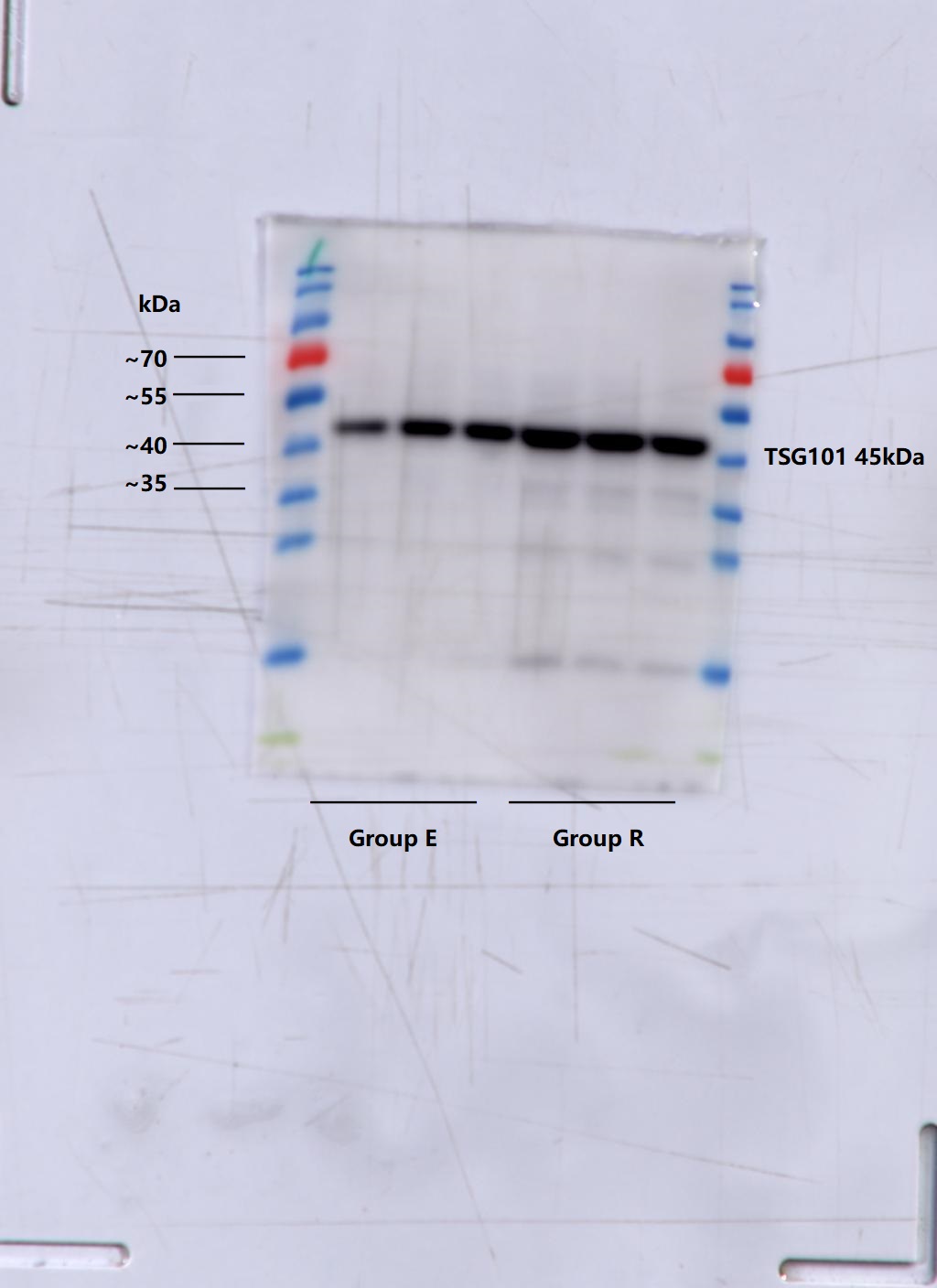

Supplement: Supplementary file 2 — Additional file 2. [file 12884_2023_5360_MOESM2_ESM.jpg]
